# Effects and Proposed Countermeasures of Abortion Bans and Restrictions on People With Uteruses and Society

**DOI:** 10.7759/cureus.29906

**Published:** 2022-10-04

**Authors:** Efe S Disi, Oluwateniayo O Okpaise, Mary-Amadeus U Akpanobong, Sofiat O Eyinfunjowo, Stephanie A Ukwandu, Magdalene O Alabintei

**Affiliations:** 1 College of Medicine, Madonna University, Elele, NGA; 2 Biology, University of Kentucky, Lexington, USA; 3 Acute Medicine, Medway Maritime Hospital, Gillingham, GBR; 4 Medicine, All Saints University Dominica, Roseau, DMA; 5 Medicine and Surgery, Olabisi Onabanjo University, Sagamu, NGA; 6 Obstetrics and Gynecology, Federal Medical Center, Yenagoa, NGA

**Keywords:** abortion 2022, abortion, abortion ban and restrictions, maternal health care, fetal anomalies, roe v. wade, illegal abortion, therapeutic abortion, abortion laws and society, abortion grounds

## Abstract

With the recent overturning of Roe V. Wade by the Supreme Court, access to abortions in many regions across the United States will become very limited as laws regarding fetal termination will be determined by state legislators rather than on a federal level. This article highlights the effects of Roe V. Wade’s abolishment on individuals that can get pregnant, how unwanted pregnancies will affect society in general, and reasonable steps forward following the ban. We conducted an electronic search using PubMed, Google, and Google Scholar. The search was retrospective, and the preliminary results focused on articles about the rationale behind pregnancy termination and the overall effects of abortion and the ban. Review papers, original papers, and newspaper articles were eligible for use. Sample size and region of publication were not exclusionary criteria. Each author independently reviewed and extracted data to write up each assigned section, and group collaborations occurred to create the final draft. Out of the 93 resources reviewed, 32 sources were deemed eligible and used in this article. These resources included 23 journal articles, eight websites, and one book.. The data gathered showed that while abortions have many potential complications even when performed under regulated conditions, taking away the choice of those with a uterus is also not without consequence. The economic, familial, and societal implications should be considered moving forward as safety nets will need to be implemented for people with uterus and children involved.

## Editorial

Introduction

Abortion (also known as the termination of pregnancy), is one of the American females' most common surgical procedures. The recent overturn of Roe V. Wade in the United States on June 24, 2022, to ban abortion has led to many heated discussions. Before the overturn, the Roe V. Wade ruling allowed for unopposed access to abortion in the first trimester. However, it allowed states to enact some regulations in the second and third trimesters. Following the overturn, several states have enacted abortion bans or restrictions.

This article points out the common reasons for abortion, the possible consequences of having an abortion, and the probable effects of an abortion ban or restriction on individuals with uterus and on society. It is good to know that the restriction or ban of abortion would save the lives of so many unborn children, yet it is not without consequences. This article also addresses countermeasures that should be implemented to buffer some of the possible negative impacts on people and society following the effects of a ban or restriction.

Reasons for abortion

The reasons for an abortion range from genetic maldevelopments to socioeconomic standing. In a study carried out by Biggs et al., financial reason was the most frequently mentioned by 40%, followed by appropriate timing (36%), and around 31% (one-third) of respondents gave partner-related reasons [1]. The need to focus on other children was mentioned by 29%, while 20% reported that having the child would interfere with their future goals and opportunities [1]. Of the respondents, 19% reported that it was due to emotional or mental unpreparedness [1]. In addition, 12% mentioned health-related reasons such as concern for their health, 6% mentioned the health of the fetus, 5% mentioned drug, tobacco, or alcohol use, and 1% mentioned non-illicit prescription drug or birth control use [1].

There are several reasons listed above, but we will touch on some of the medical health aspects. Regarding the health of the fetus, fetal incompatibility with life and anomalies are one of the underlining reasons for abortion [2]. Aborting such a fetus reduces the rigors that come with providing supportive care and healthcare. Among the fetal-related causes for abortion, a study by Mahdavi et al. listed nervous system malformations (26.4%), chromosomal abnormalities (18.4%), hydrops fetalis (13.7%), and musculoskeletal anomalies (9.9%) as the most common reasons [3].

Asides from fetal health-related reasons, there are also maternal health-related reasons. Some health conditions make mothers unfit to carry a pregnancy to term, which can lead to what is called "therapeutic abortion." In the same study by Mahdavi et al., the most common maternal health-related reasons for abortion were circulatory system diseases (43.9%), neoplasms (13.4%), and genitourinary system diseases (9.9%) [3].

Lastly, almost 3 million women in the United States have experienced rape-related pregnancy during their lifetime [4]. Sexual violence occurs on a continuum, has devastating impacts on victims, and contributes to psychiatric disorders. Although having an abortion may not remove the initial trauma, victims of sexual assault should have the right to a full range of emotional, medical, and legal support options.

Effects of abortion 

There are numerous reasons for getting an abortion; however, the decision to have an abortion is not without residual physical and psychological effects.

Pregnant Person: Physical and Mental Effects

While the safety of abortions has increased in the United States since the implementation of Roe V. Wade, a 0.5% complication rate is seen with first-trimester abortions, which doubles in the second trimester [5]. The risks of abortion-related complications are directly proportional to maternal age, gestational age, and the number of parity [5].

The physical adverse effects of abortion can be classified based on three mechanisms: infection resulting from improper sterilization, incomplete evacuation of fetal tissues, and iatrogenic injury to the reproductive tract [6]. While infections resulting from regulated abortions are relatively rare, human error plays a significant role, as errors such as insufficient handwashing, improper surgical glove use, and sterilization of the surgical field can result in complications [5,6]. Complications such as endometritis, cervicitis, and maternal sepsis can occur due to an iatrogenic error, which can cause pelvic inflammatory disease and lead to secondary infertility.

Incomplete or failed abortions are more common when procedures occur earlier in the gestational periods. This complication means that the entirety of the products of conception was not removed from the uterus [6]. Genitourinary tract lesions can occur during fetal termination, depending on the devices used during the procedure. While cervical lacerations are common, severe injuries to adjacent organs such as the bowel or bladder can also occur [6].

Just as a person can have a myriad of reasons for choosing to have an abortion, their emotional response following the procedure can also be varied. Depending on an individual's reasoning, emotions can range from relief and happiness to sadness and regret. Pregnancy loss is generally associated with alterations in the expecting individuals' hormonal cycle; thus, negative feelings can be partly attributed to these imbalances [7].

Asides from the possible cause of psychological changes emanating from hormonal imbalance after pregnancy loss, psychological changes after an abortion can also be influenced mainly by the socioreligious status of the society and one's moral background. Aspects such as relationships, societal stigma, and religion can add to the feeling of isolation a pregnant individual may feel, increasing their feelings of loneliness and even depression [8]. A study conducted by Rocca et al. revealed that when it came to unwanted pregnancies, women tended to attribute negative feelings toward the pregnancy rather than the abortion itself [9]. These women felt relief following the procedure; this shows that a broad spectrum of emotional states can exist following the elective termination of pregnancy. Despite the varied emotions women experience in the weeks following their abortion, most report that the decision to end their pregnancy was the right one; however, it is still essential to support struggling women, such as through counseling [9].

Effects on Relationships

Abortion not only affects the individual getting the procedure but can also affect their standing in both familial and societal settings [10]. Marital strain following an elective abortion is not uncommon due to both the experience of the abortion and the response of the pregnant individual's partner. As stated previously, hormonal imbalance can occur due to the disruption of the pregnancy. This could be expressed as emotional distress in which the other partner may be blamed for the unwanted pregnancy or maternal depression if the abortion was coerced, forced, or against the affected partner's will [10].

A reduction in libido following the procedure is not uncommon [11] and can also be a complication, leading to the development of a rift between partners. Depending on the factors surrounding the pregnant individual's decision to abort their pregnancy, the period following an abortion can make emotional attachments and bonds difficult for some women [10].

Effects of the abortion ban and restriction on people 

Not only can the process of receiving an abortion affect people with uterus physically and mentally, but being denied one can also have ramifications. A five-year research project known as the Turnaway Study showed that women who sought abortions but were denied one had more complications in both the gestational and post-gestational periods than their counterparts who got the procedure [12]. These women reported higher rates of chronic headaches, gestational hypertension, seizures, and hemorrhage in the postpartum period. They can also have high rates of depression, low self-esteem, stress, and anxiety. Another finding of the Turnaway Study was that being denied an abortion increased the likelihood that women would remain in abusive relationships at higher rates than their cohorts who had abortion access [12].

Maternal health is an area that an abortion ban or restriction can negatively impact. Maternal health refers to the health of people with uterus during pregnancy, childbirth, and the postnatal period. Each stage should be a positive experience, ensuring that people with uterus and their babies reach their full potential for health and well-being. A stop to preventable maternal death is crucial, and at the same time, simply surviving pregnancy and childbirth should not be the only marker of successful maternal health care. Maternal deaths from unsafe abortion continue to occur globally, with exceptionally high rates in Sub-Saharan Africa, where most abortions are classified as unsafe. Maternal death reviews are a practical part of cohesive strategies to prevent future deaths while abortion remains illegal. A case series from Uganda showed that most maternal deaths owing to unsafe abortion were found to be preventable and concluded that postabortion care is part of essential emergency medical care and should be provided with high standards, especially in areas where there is limited or no legal access to abortion care [13]. The abortion ban and restriction may lead to a rise in maternal mortality in the United States, especially in people of color [14]. In 2020, the maternal mortality rate in the United States was 23.8 deaths per 100,000 live births, with women of color, particularly black women, being the most affected [15]. These numbers will likely increase in the coming years, as pregnant individuals who feel trapped in their pregnancy could try potentially dangerous alternatives to end their pregnancy. The Turnaway Study has highlighted that roughly 21% of women in the United States will participate in unsafe abortions. Partaking in these unregulated procedures will increase abortion-related death by up to 33%, particularly among underserved populations such as African-Americans and other minority groups in the United States [16]. When abortion is done safely, patients can return to their physicians for check-ups, but if unsafe abortions are performed, these individuals have no proper healthcare preventing medical complications such as sepsis, hemorrhage, pelvic injuries, and toxic exposure.

Many mothers who choose to undergo an abortion state that they choose to undergo an abortion because they already have children and do not wish to impact their lives negatively. The Turnaway Study showed that being denied an abortion affects not only the mother but also her relationship with already existing children [13]. Results showed that children under the age of 5 born to patients who were denied abortions were less likely to meet developmental milestones at an appropriate timeline and were more likely to experience stress related to poverty [13].

It is important to note that the number of children abandoned and in foster homes will likely rise, leading to a challenging and poor upbringing. Research conducted by Foster et al. found that unwanted children were raised in homes with incomes 31% lower than the federal poverty level and did not have enough funds for basic needs [17]. They also found that the children had poor maternal bonds and were more likely to have injuries, neglect, or abuse [17].

Effects of abortion ban and restriction on the society

An individual's ability to make her own reproductive choices positively affects her society. Having reproductive autonomy allows individuals with uterus to choose when they want to get pregnant and how many children they would like overall [18]. This freedom of choice can allow these individuals to enter and finish programs of higher educational status at the same rate as their male counterparts. The degrees gained from these programs give individuals with uterus a more stable footing when entering the workforce, as they no longer need to decide between having a career and starting a family [18]. With access to abortion, governmental funding to support individuals with uteruses with an unwanted or unplanned pregnancy can be diverted to other aspects of society, such as infrastructure or healthcare. Giving persons with uteruses the ability to plan their pregnancies around their late 20s to early 30s can aid in mitigating familial socioeconomic and educational gaps, giving future generations the ability to reach heights that would have been improbable without abortion access [18].

Abortion is not evenly distributed among the population. According to data from the Centers for Disease Control and Prevention (CDC), black women are five times more likely to have an abortion than white women, and Latin women are twice as likely as whites [19]. Seventy-five percent of people who have abortions have low incomes or live in poverty and more than sixty percent have other children [20]. Given the lack of pregnancy and parental support in the United States (that is, no paid parental leave, limited choices for healthcare coverage, no support for childcare), forcing those on low incomes to choose between paying to travel to get an abortion against adding another child to their lives will place many families in worsening poverty [20].

Legalization of abortion has been linked to crime reduction, such as in a study conducted by Donohue and Levitt, it was shown that crime rates across states appeared to have dropped as a result of Roe V. Wade [21]. The likely reason for this outcome is that more children from unwanted pregnancies born in low-income households in underprivileged communities before the legalization of abortion were at a greater risk of engaging in crime as adults. Abortion liberalization reduced the incidence of such unwanted births, with a subsequent reduction in crime years later coinciding with the period when the babies would have been adults. However, the finding may not apply to other countries. For example, Buonanno et al. [22] showed that in Europe, abortion restriction is not associated with reduced crime, most likely because solid welfare systems and family ties minimize the risk that unwanted childbearing results in greater crime [22].

Next steps following an abortion ban or restriction

In the wake of an abortion ban or restriction, preventing unwanted pregnancies becomes more crucial, and contraception becomes an essential means of preventing unwanted pregnancies. Making contraception readily available, that is, over the counter, available without a prescription, and affordable to every person of all socioeconomic status, would be very helpful, especially among the poor. Not only would it help reduce financial burdens on an individual level, but it would also, on a larger scale, reduce the poverty level of society. Contraceptives come with associated risks like any other medications. For example, combined oral contraceptives (COCPs) are known to increase the risks of having a stroke in persons with uterus who are obese and/or are smokers, which calls for screening before administering. However, several studies have shown self-screening as effective as physician screening [23-25], making over-the-counter COCPs more feasible. Other contraceptives administered through routes other than orally and with the assistance of a physician should be made affordable with little or no out-of-pocket expenses. Permanent birth controls such as tubal ligations and vasectomies may also be encouraged in individuals who can afford it and are satisfied with their family size.

The rate of teenage pregnancy in the United States has declined over the past decade [26]; however, the United States remains one of the countries with the highest teenage pregnancy rates in the world, and approximately 30% of these pregnancies end in abortions [27]. With the restrictions and bans on abortion, reducing the rate of adolescent pregnancy is essential, especially among the low socioeconomic groups. One way to reduce teenage pregnancy rates in the United States is to look at countries with low teenage pregnancy rates and examine the measures used and their impact on birth rates. For example, countries like the Netherlands have one of the lowest birth and abortion rates among teenagers [27,28]. Studies in the Netherlands showed a more open attitude towards sexual relationships and sex education (including contraception). The educational system and parents introduce their children to sex education and relationships at an early age [28]. Furthermore, Dutch teens were found to be more open to discussing contraception options with their partners [28]. The openness, early introduction, and supportive atmosphere associated with sex education, including contraceptive use, appear to have played a significant role in the effectiveness of sex education on birth and abortion rates among their teenagers. Abstinence-only sex education has been the prominent form of sex education in the United States since the 1990s. Overall, 25% of the decline in teenage pregnancy between 1988 and 1995 in the United States due to reduced sexual activities was likely the effect of abstinence-only sex education; however, the remaining 75% resulted from the effective use of contraceptives [26]. Comprehensive sex education focuses on abstinence and includes discussions surrounding contraception, safe relationships, age of consent, prevention of sexual diseases, and pregnancy outcome options. It is likely to be more effective as it is similar to the type of education used in countries with low teenage birth and abortion rates. Also, studies have shown it to be more effective than abstinence-only sex education [29]. Currently, only about 21 states in the U.S. mandate sex education that includes discussions surrounding contraception [30]. Requiring a comprehensive sex education in the majority, if not all, of states in the United States could help reduce the financial and socioeconomic burdens that come with unwanted teenage pregnancies in the event of a ban and restriction on abortion.

If an unwanted pregnancy is inevitable, more centers that render services to pregnant people, such as pregnancy resource centers, should be implemented and funded. Such centers should provide social and financial support and free access to antenatal care, especially to pregnant teens, people with meager incomes, and the uninsured. These centers should also provide parenting classes to pregnant persons and their partners to effectively equip them to raise their unborn children. Regarding children born with special needs, more centers that render services such as learning centers for the mentally and physically challenged should be implemented. There should also be free access to healthcare for children who depend on medical treatments most of their lives to reduce the financial costs of caring for them.

It is crucial to make the pregnant individuals aware that they have other choices aside from parenting. Awareness could be made through the media, healthcare workers, medical outreach programs, comprehensive sex education at schools, and pregnancy resource centers. Pregnancy resource centers should offer the option of adoption and connect them to adoption agencies when a person is unwilling or incapable of raising the child. One could choose from different types of adoption depending on varying degrees of contact with the child. There are open, semi-open, and closed types of adoptions. The birth mother is allowed to select the adoptive family in all types; however, open adoption allows for the most contact with the child, whereas semi-open adoption limits direct contact but receives information about the child [31]. In a closed adoption, there is no contact, and the birth mother's identity is kept confidential [31]. Helping them know they can control the level of involvement in their child's upbringing can be reassuring.

When the right to choose to terminate a pregnancy is taken away from people with uterus, it seems reasonable to empower them in ways that will allow them to cope. Empowering people with uterus through providing jobs, mandating equal pay, and childcare, especially within the workplace, is paramount, as one of the significant reasons for unwanted pregnancies is financial constraints (unemployment and low-paying jobs), or lack of reliable and affordable childcare.

Lastly, the healthcare system should be equipped and anticipate more cases of failed abortion and abortion complications from unsafe abortion practices, as unsafe abortion practices and abortion deaths would be on the rise with abortion restrictions. The Cates-Rochet study examined the effects of legalizing abortion on abortion deaths and illegal abortions between 1972 and 1975, finding a decrease in illegal abortions from 130,000 to 17,000 and a decrease in abortion deaths from 39 to 5 in that same period [32]. It is evident that there will be more cases of failed abortions and abortion deaths from illegal abortions in the event of abortion restrictions and bans.

Conclusion

All humans are considered moral beings. We inherently know wrong and right. Most societies instill morality in their laws regardless of religion. People are expected to make choices that not only impact themselves positively but also not harm others simultaneously. It is reasonable to consider the most beneficial in decision-making, especially when both sides can be negatively impacted. The question remains, which is more important or beneficial in the long run, allowing a life into the world with the challenges that may come with it, versus preventing one based on finances, career, health, and relationship problems? The answer to this question can be predicted based on different factors but is not certain.
